# Astrocyte calcium waves propagate proximally by gap junction and distally by extracellular diffusion of ATP released from volume-regulated anion channels

**DOI:** 10.1038/s41598-017-13243-0

**Published:** 2017-10-13

**Authors:** Yuki Fujii, Shohei Maekawa, Mitsuhiro Morita

**Affiliations:** Kobe University Graduate School of Science, Department of Biology, Kobe, 657-8501 Japan

## Abstract

Wave-like propagation of [Ca^2+^]_i_ increases is a remarkable intercellular communication characteristic in astrocyte networks, intercalating neural circuits and vasculature. Mechanically-induced [Ca^2+^]_i_ increases and their subsequent propagation to neighboring astrocytes in culture is a classical model of astrocyte calcium wave and is known to be mediated by gap junction and extracellular ATP, but the role of each pathway remains unclear. Pharmacologic analysis of time-dependent distribution of [Ca^2+^]_i_ revealed three distinct [Ca^2+^]_i_ increases, the largest being in stimulated cells independent of extracellular Ca^2+^ and inositol 1,4,5-trisphosphate-induced Ca^2+^ release. In addition, persistent [Ca^2+^]_i_ increases were found to propagate rapidly via gap junctions in the proximal region, and transient [Ca^2+^]_i_ increases were found to propagate slowly via extracellular ATP in the distal region. Simultaneous imaging of astrocyte [Ca^2+^]_i_ and extracellular ATP, the latter of which was measured by an ATP sniffing cell, revealed that ATP was released within the proximal region by volume-regulated anion channel in a [Ca^2+^]_i_ independent manner. This detailed analysis of a classical model is the first to address the different contributions of two major pathways of calcium waves, gap junctions and extracellular ATP.

## Introduction

Deeper understanding of the mechanisms underlying the spatio-temporal diversity and complexity of [Ca^2+^]_i_ increases in astrocytes is crucial for exploring the physiological and pathological functions of this glial cell population. Calcium waves are a remarkable aspect of [Ca^2+^]_i_ dynamics in astrocytes, and a unique type of intercellular communication in astrocyte networks, intercalating neuronal circuitries and vasculature. Various pharmacologic and physical stimuli have been found to induce [Ca^2+^]_i_ increases propagating between astrocytes in cell cultures^1,2^, in brain slices^3,4^, and in other *in vivo* preparations^5,6^. These calcium waves are regarded as transmitting physiologic and pathologic signals within the brain, because they influence the activities of adjacent neurons^7,8^, microglia^9^, and endothelial cells^10^. Furthermore, recent studies have demonstrated the involvement of astrocyte networks and calcium waves in regulating neuronal activities^11^ and neurological diseases^12,13^.

Because calcium waves can propagate between astrocytes in the absence of physical contact^14^, they are likely induced by intracellular and extracellular signals in a synergistic manner. Astrocytes are intracellularly connected via connexin channels^15^, and their transmission of Ca^2+^ and IP_3_ via gap junctions has been demonstrated experimentally and theoretically^16,17^. Moreover, astrocytes are equipped with ATP release mechanisms and ATP receptors inducing [Ca^2+^]_i_ increases^18^, and purinergic signaling has been found to be involved in calcium waves^19,20^. Furthermore, gap junction and purinergic signaling are regulated in a supplementary manner to maintain calcium waves^21^. However, the contributions of these components to the dynamics and functions of calcium waves, and the mechanisms involved in initiating [Ca^2+^]_i_ increases and release ATP in calcium waves are incompletely understood.

Theoretical^17^ and experimental^1,2^ studies have shown that calcium waves can be mechanically induced by gently touching cultured astrocytes with tips of glass pipettes. The present analysis of this classical model pharmacologically and by using an ATP sniffing cell revealed distinct [Ca^2+^]_i_ increases during calcium waves. This study was therefore designed to assess the distinct contributions of gap junction and extracellular ATP and the ATP release mechanism in calcium waves, revealing novel aspects of the diverse and complicated dynamics of astrocyte [Ca^2+^]_i_.

## Results

### Components of [Ca^2+^]_i_ increases in calcium waves

**Figure 1:**
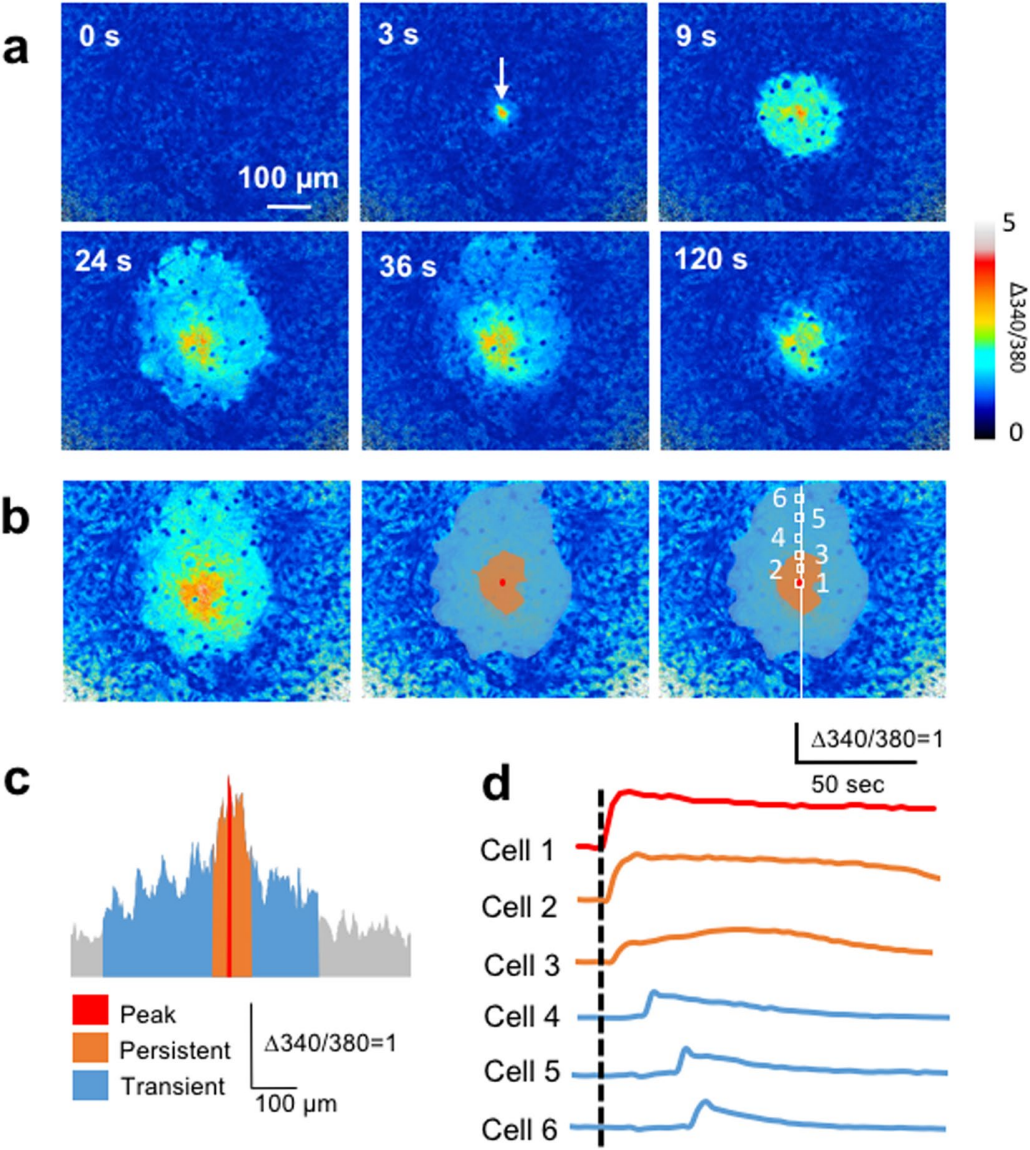
Distinct components of [Ca^2+^]_i_ increases in an astrocyte calcium wave. (**a**) Representative Fura2 ratio (Δ340/380) images of an astrocyte calcium wave 0, 3, 9, 24, 36 and 120 sec after mechanical stimulation (arrow). (**b**) Distribution of [Ca^2+^]_i_ increases during the calcium wave. The maximum [Ca^2+^]_i_ projection, in which each pixel represents the maximum Δ340/380 during the calcium wave (left). The peak [Ca^2+^]_i_ increase (red), which is the [Ca^2+^]_i_ increase in the mechanically-stimulated cell, and the persistent (orange) and transient (blue) [Ca^2+^]_i_ increases, in which the Δ340/380 ratio of each pixel sustained above and declined below 30% of the mean Δ340/380 ratio of pixels within the peak [Ca^2+^]_i_ increase until 120 sec, respectively are shown overlaid on the maximum [Ca^2+^]_i_ projection (center). A Line and cells for the analyses in (**c**) and (**d**) are indicated in the right panel. (**c**) Histogram of the maximum Δ340/380 along the line in the right panel of (**b**). (**d**) Time-dependent changes in [Ca^2+^]_i_ in cells 1–6 in the right panel of (**b**).

Figure 1a shows a representative calcium wave induced by mechanical stimulation of cultured astrocytes. The [Ca^2+^]_i_ increase in the mechanically-stimulated cell (arrow) propagated to adjacent cells, and the area of [Ca^2+^]_i_ increases reached a maximum at 24 sec. Then, [Ca^2+^]_i_ in the distal region declined to the baseline by 120 sec, whereas that proximal to the stimulated cell remained elevated for longer than 120 sec. The distribution of [Ca^2+^]_i_ increases was expressed as a maximum [Ca^2+^]_i_ projection, in which each pixel represents the maximum Δ340/380 ratio during the calcium wave (Fig. 1b left). As shown in Fig. 1b center, we defined the peak [Ca^2+^]_i_ increase (red) as [Ca^2+^]_i_ increase in the stimulated cell, and the persistent (orange) and transient (blue) [Ca^2+^]_i_ increases as [Ca^2+^]_i_ increases sustained and declined until 120 sec, respectively. The appropriateness of 120 sec was clarified later. The histogram of maximum [Ca^2+^]_i_ increases along a line in Fig. 1b right, shows that the peak [Ca^2+^]_i_ increase was the largest [Ca^2+^]_i_ increase during the calcium wave, and the persistent [Ca^2+^]_i_ increases were larger than the transient [Ca^2+^]_i_ increases (Fig. 1c). The [Ca^2+^]_i_ increases of individual cells in the region of the peak and persistent [Ca^2+^]_i_ increases (Cell 1–3) were persistent, whereas those in the region of transient [Ca^2+^]_i_ increases (Cell 4–6) were transient (Fig. 1d). These findings suggested that the peak, persistent and transient [Ca^2+^]_i_ increases were distinct components of the same calcium wave.

**Figure 2:**
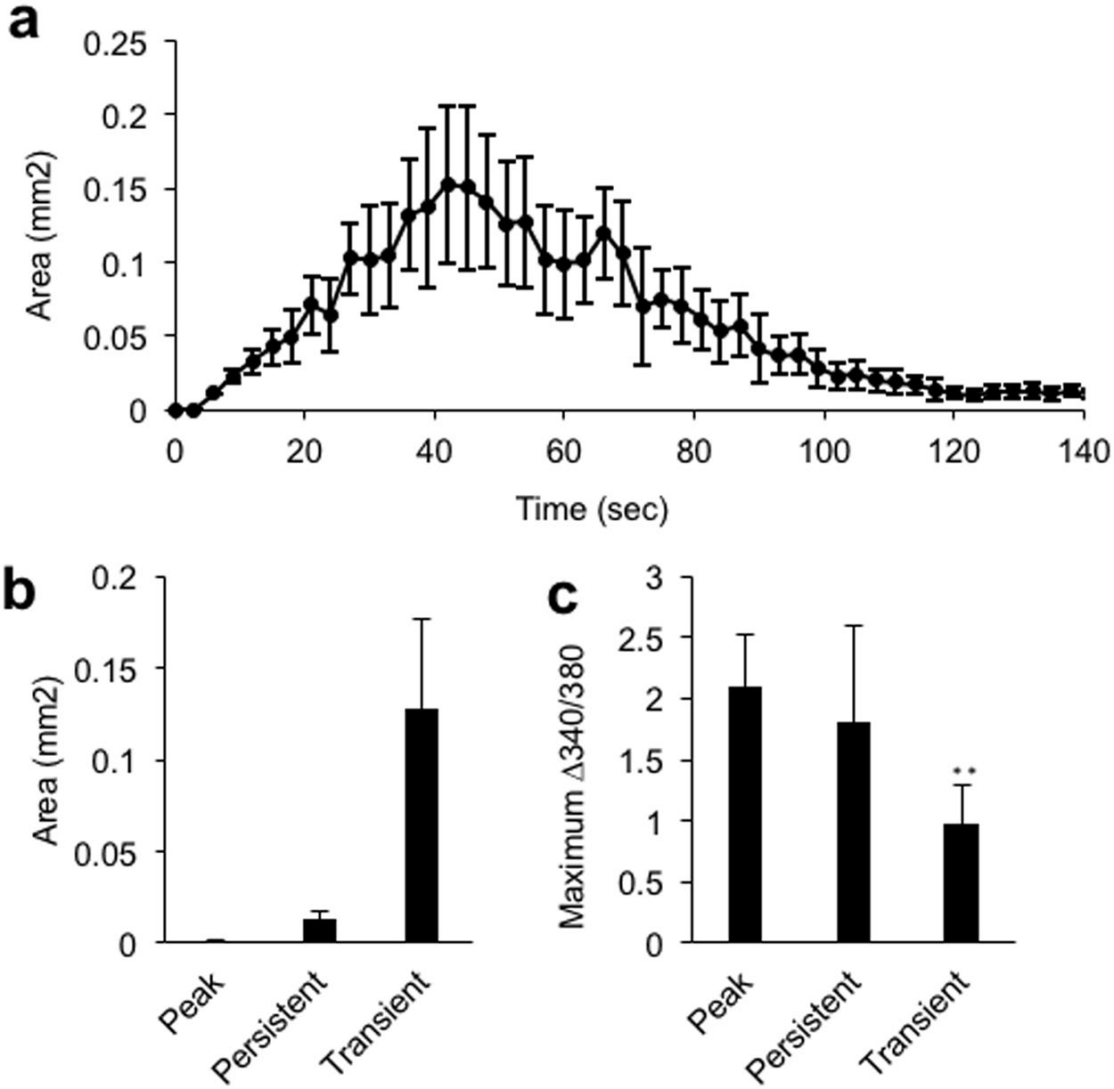
Time-dependent changes of area of [Ca^2+^]_i_ increase during calcium waves. Calcium waves (n = 10 experiments) were subjected to the following analysis. The area of [Ca^2+^]_i_ increase was calculated from the total number of pixels whose Δ340/380 ratio was above 30% of that of the peak [Ca^2+^]_i_ increase to distinguish the transient from the persistent increases, Fig. 1. (**a**) Averaged area of [Ca^2+^]_i_ increases at each time point. Maximum areas (**b**) and maximum [Ca^2+^]_i_ increases (**c**) of calcium wave components. **p < 0.01 versus Peak, Tukey post hoc test after one-way ANOVA.

The time dependent changes of areas of [Ca^2+^]_i_ increases were further analyzed by averaging 10 calcium waves. The area of [Ca^2+^]_i_ increases peaked at 40 sec and declined to become constant at 120 sec as shown in Fig. 2a. Thus, it is appropriate to define the persistent [Ca^2+^]_i_ increase depending on the area of [Ca^2+^]_i_ increase at 120 sec. The areas of peak, persistent and transient [Ca^2+^]_i_ increases were 1133 ± 453 μm^2^, 13069 ± 4574 μm^2^ and 127633 ± 51000 μm^2^, respectively (Fig. 2b), equivalent to circles with radii of 19 μm, 65 μm and 201 μm respectively. The averaged maximum [Ca^2+^]_i_ in the areas of peak and persistent [Ca^2+^]_i_ increases did not significantly differ, but that in the area of transient [Ca^2+^]_i_ increases was significantly smaller than that in the area of peak [Ca^2+^]_i_ increases by 46% as shown in Fig. 2c. These results further confirm the difference between transient and persistent [Ca^2+^]_i_ increases.

### Mechanisms increasing [Ca^2+^]_i_ in calcium waves

**Figure 3:**
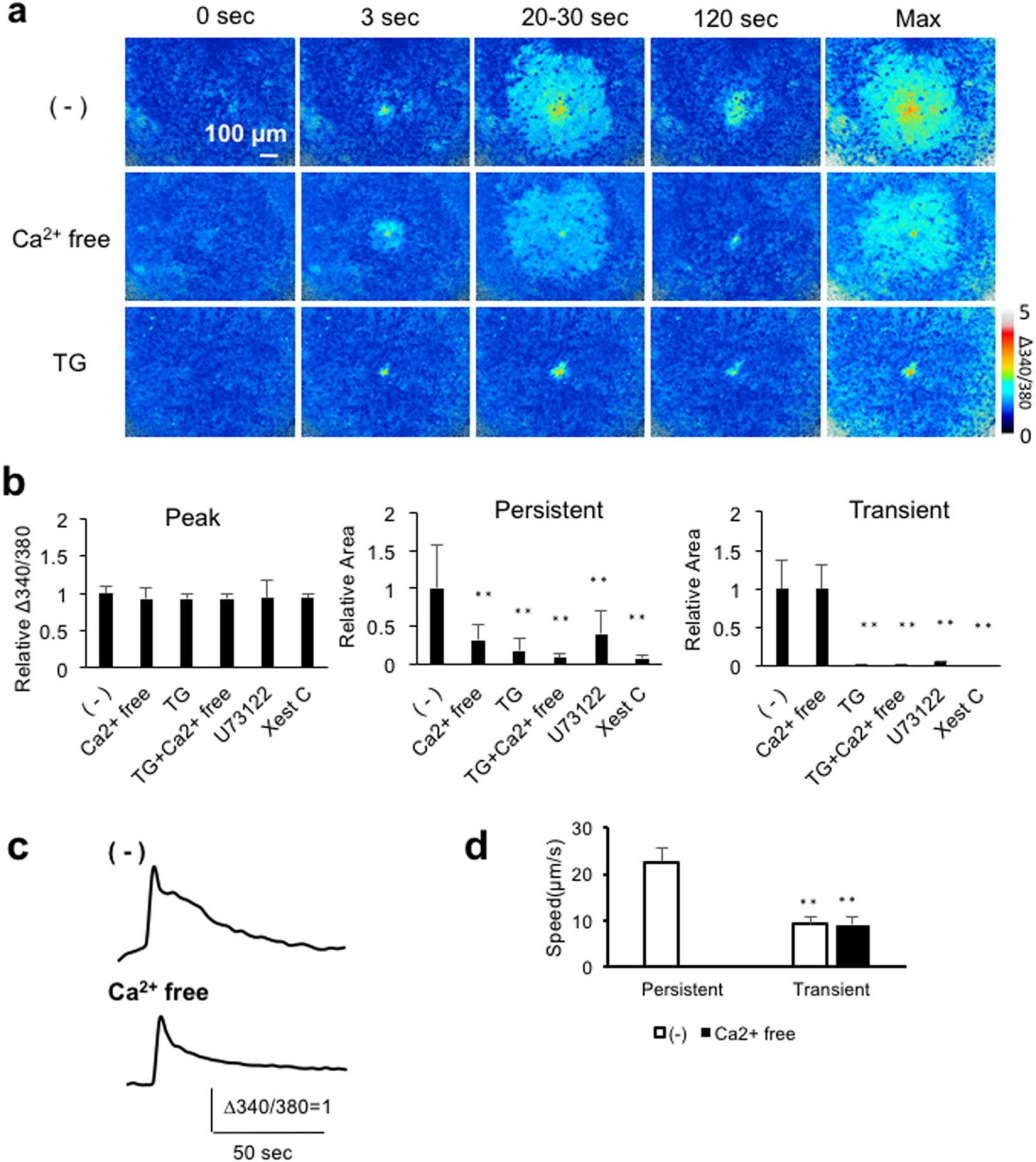
Pharmacologic characterization of [Ca^2+^]_i_ increases during calcium waves. Astrocytes were treated with Ca^2+^-free extracellular solution (Ca^2+^-free), 1 μM thapsigargin (TG), 10 μM U73122 or 20 μM Xestospongin C (Xest C) for 10 min prior to mechanical stimulation. (**a**) Representative calcium waves of control (−), Ca^2+^-free and TG-treated cells. Baseline [Ca^2+^]_i_ (0 sec), initial [Ca^2+^]_i_ increase (3 sec), maximally-propagated [Ca^2+^]_i_ increase (20–30 sec), sustained [Ca^2+^]_i_ increase (120 sec) and the maximum [Ca^2+^]_i_ projection (Max). (**b**) Amplitudes of the peak [Ca^2+^]_i_ increases (Peak Δ340/380, left), and areas of the persistent (center) and transient (right) [Ca^2+^]_i_ increases normalized to control. n = 8 experiments; **p < 0.01 versus (−), Bonferroni’s multiple comparison test after one-way ANOVA. (**c**) Representative transient [Ca^2+^]_i_ increases in individual cells of control and Ca^2+^-free treated cultures. (**d**) Effects of extracellular Ca^2+^ on the velocities of calcium wave propagations in the regions of persistent or transient [Ca^2+^]_i_ increases. n = 6 experiments; **p < 0.01 versus (−) in the region of persistent [Ca^2+^]_i_ increases, (−), Bonferroni’s multiple comparison test after one-way ANOVA.

The mechanisms underlying the [Ca^2+^]_i_ increases during calcium waves were pharmacologically examined by omitting extracellular Ca^2+^ (Ca^2+^-free), which inhibits Ca^2+^ influx; and by treating cells with 1 μM thapsigargin (TG), 10 μM U73122 and 20 μM Xestospongin C (Xest C), which inhibit, calcium storage, inositol1,4,5-trisphosphate (IP_3_) production and IP_3_ receptor, respectively. Figure 3a shows representative calcium waves of control (−), Ca^2+^-free and TG-treated cells. Omitting extracellular Ca^2+^ was found to eliminate the persistent [Ca^2+^]_i_ increases without affecting the peak [Ca^2+^]_i_ increase or propagation of the calcium wave, whereas treating with thapsigargin abolished the calcium wave without affecting the peak [Ca^2+^]_i_ increase. Figure 3b shows quantitative analyses of the calcium wave components. Amplitudes of the peak [Ca^2+^]_i_ increases were not significantly affected by any pharmacologic treatment, indicating that the peak [Ca^2+^]_i_ increases were not mediated by Ca^2+^influx or IP_3_-induced Ca^2+^release (IICR), and that alterations of calcium waves described below did not reflect the difference of initial [Ca^2+^]_i_ increases before propagations. In contrast, incubation of cells with Ca^2+^-free medium, TG, TG + Ca^2+^-free medium, U73122, and Xest C resulted in significant reductions in areas of the persistent [Ca^2+^]_i_ increases to 32%, 18%, 9%, 40%, and 8%, respectively. The elimination of persistent [Ca^2+^]_i_ increases was partial in some experiments of Ca^2+^-free group, presumably due to insufficient reduction of extracellular Ca^2+^ concentration in the nominally Ca^2+^-free condition. Although Ca^2+^-free medium had no effect on areas of the transient [Ca^2+^]_i_ increases, the latter was significantly reduced by TG, TG + Ca^2+^-free, U73122 and Xest C to 2%, 2%, 5% and 1%, respectively. These findings indicate that the persistent [Ca^2+^]_i_ increase is maintained synergistically by Ca^2+^influx and IICR, whereas the transient [Ca^2+^]_i_ increases are mediated by IICR alone.

The effects of omitting extracellular Ca^2+^ on calcium waves were further analyzed. A representative [Ca^2+^]_i_ increase in an individual cell treated with Ca^2+^-free medium was transient similar to that in the distal region of control (Fig. 3c). The velocity of calcium wave in the Ca^2+^-free group (9.1 ± 1.8 μm/sec) were similar to that of the transient [Ca^2+^]_i_ increases in the control group (9.3 ± 1.6 μm/sec), with both being significantly lower than the velocity of the persistent [Ca^2+^]_i_ increase in the control group (22.6 ± 2.9 μm/sec) (Fig. 3d). These results indicate that extracellular Ca^2+^ does not affect the propagations of the transient [Ca^2+^]_i_ increases.

### Mechanisms of calcium wave propagation

**Figure 4:**
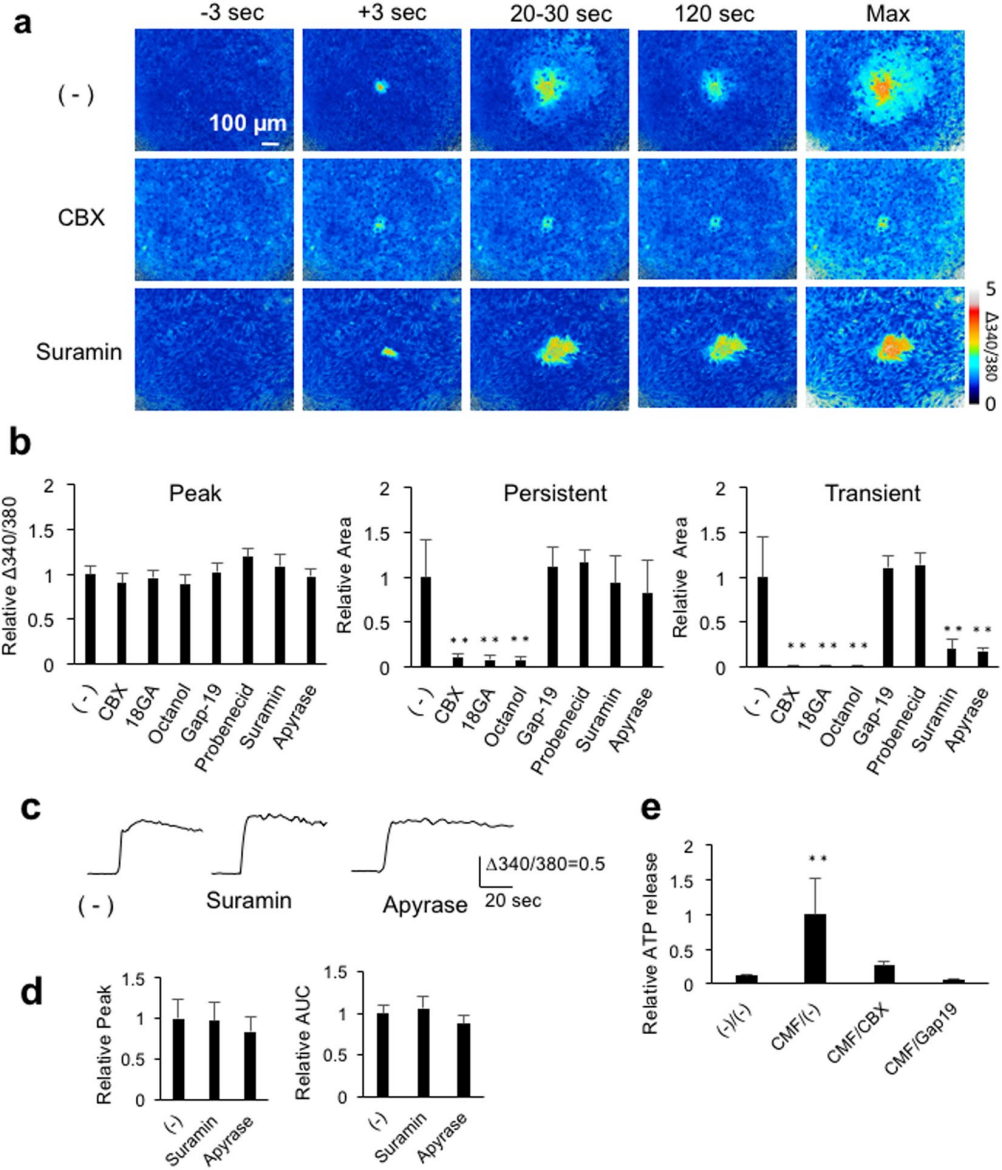
Pharmacologic characterization of calcium wave propagation. Astrocytes were treated with 100 μM carbenoxolone (CBX), 100 μM 18β-glycyrrhetinic acid (18βGA), 1 mM octanol, 300 μM gap-19, 1 mM probenecid, 100 μM suramin or 15 unit/ml apyrase for 10 min prior to mechanical stimulation. (**a**) Representative calcium waves of control (−), CBX- and suramin-treated cells. (**b**) Amplitudes of the peak [Ca^2+^]_i_ increases, and areas of the persistent and transient [Ca^2+^]_i_ increases normalized to control. n = 8 experiments; **p < 0.01 versus (−), Bonferroni’s multiple comparison test after one-way ANOVA. (**c**) Representative persistent [Ca^2+^]_i_ increases in individual cells of control, suramin- and apyrase-treated cultures. (**d**) Quantification of [Ca^2+^]_i_ increases in the region of persistent [Ca^2+^]_i_ increases, as shown by the area under the curve (AUC) and the peak amplitude (Peak) for 70 sec after mechanical stimulation, normalized to control. n = 15 cells from three independent experiments. No significant difference was observed by one-way ANOVA. (**e**) Effects of CBX and gap-19 on ATP release via gap junction hemichannels activated by treatment with calcium-magnesium free (CMF) medium. ATP in the extracellular solution 10 min after pharmacologic treatment was measured by luciferase assays and normalized to CMF/(−). n = 3 wells; **p < 0.0101 versus (−)/(−), Bonferroni’s multiple comparison test after one-way ANOVA.

The mechanisms underlying calcium wave propagation were pharmacologically examined by treating cells with the gap junction blockers, 100 μM carbenoxolone (CBX), 100 μM 18β-glycyrrhetinic acid (18GA) and 1 mM octanol; a blocker of gap junction hemichannels containing connexin 43 (Cx43), which is a major connexin subtype in cultured astrocytes^22^, 300 μM gap-19^23^; a pannexin hemichannel blocker, 1 mM Probenecid; an ATP receptor antagonist, 100 μM suramin; and an ATP degrading enzyme, 15 unit/ml apyrase. Figure 4a shows representative calcium waves of control, CBX- and suramin-treated cells. These results indicate that CBX abolishes calcium waves without affecting the peak [Ca^2+^]_i_ increases, whereas suramin eliminates the transient [Ca^2+^]_i_ increases without affecting the peak or the persistent [Ca^2+^]_i_ increases. Figure 4b shows quantitative analyses of the calcium wave components. The peak [Ca^2+^]_i_ increases were not significantly affected by any pharmacologic treatments, indicating that they do not involve either gap junction or purinergic signaling. In contrast, CBX, 18GA and octanol significantly reduced areas of the persistent [Ca^2+^]_i_ increases, to 10%, 8% and 7%, respectively, and reduced areas of the transient [Ca^2+^]_i_ increases to 1%, 1% or 0.8%, respectively, indicating that gap junctions are essential for calcium waves. Gap-19 and probenecid did not affect either the persistent or transient [Ca^2+^]_i_ increases, indicating that connexin and pannexin hemichannels are not involved in calcium waves. Suramin and apyrase had no effect on areas of the persistent [Ca^2+^]_i_ increases, but significantly reduced areas of the transient [Ca^2+^]_i_ increases to 20% or 18%, respectively, suggesting that purinergic signaling is involved in the generation and/or propagation of transient [Ca^2+^]_i_.

The effects of purinergic inhibitors on the persistent [Ca^2+^]_i_ increases were further analyzed. Individual astrocytes showed persistent [Ca^2+^]_i_ increases, regardless of the presence of purinergic inhibitors (Fig. 4c). The extent of [Ca^2+^]_i_ increases, expressed as peak amplitude (peak) or area under the curve (AUC), was not affected by these purinergic inhibitors (Fig. 4d), indicating that purinergic signaling is not involved in the persistent [Ca^2+^]_i_ increases.

The inhibition of astrocyte gap junction hemichannels by gap-19 was confirmed by measuring ATP release, using luciferase assays, following treatment in Ca^2+^ and Mg^2+^ free (CMF) medium, which is commonly used to induce the release of macromolecules from gap junction hemichannels^24^. As expected, CMF enhanced extracellular ATP, but this increase was significantly reduced to 4% and 10% by CBX and gap-19, respectively (Fig. 4e), indicating that gap-19 potently inhibits astrocyte gap junction hemichannels. These findings confirmed that calcium waves do not involve ATP release via gap junction hemichannels.

### ATP release mechanisms in calcium waves

**Figure 5:**
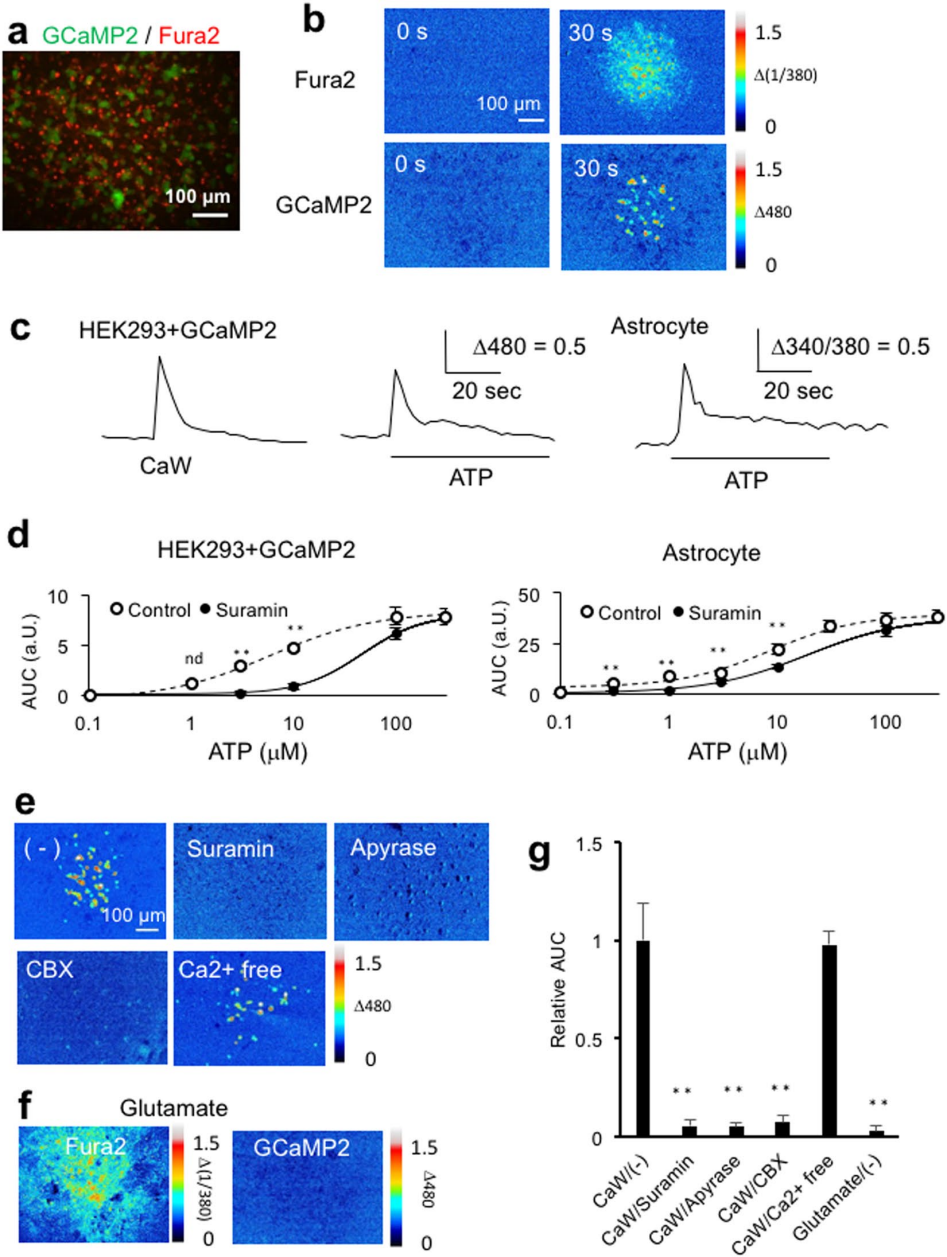
Pharmacologic characterizations of ATP release during calcium waves using HEK293 + GCaMP2 cells. Calcium waves and ATP release were simultaneously imaged as fluorescence changes in Fura2-loaded astrocytes and in HEK293 + GCaMP2 cells placed on the astrocytes, respectively. (**a**) Representative fluorescence images of Fura2-loaded astrocytes (Ex/Em = 380/535, red) and HEK293 + GCaMP2 cells (Ex/Em = 480/535, green). (**b**) Representative images of [Ca^2+^]_i_ increases in astrocytes (Fura2) and HEK293 + GCaMP2 cells (GCaMP2) 0 and 30 sec after mechanical stimulation. (**c**) Representative [Ca^2+^]_i_ increases in individual HEK293 + GCaMP2 cells and astrocyte during a calcium wave (CaW) and following ATP treatment (10 μM, bar). Cells were stimulated to induce calcium waves and then treated with ATP by bath loading. ATP responses of astrocytes and HEK293 + GCaMP were indistinguishable between areas with or without the calcium wave. (**d**) Concentration dependence of ATP-induced [Ca^2+^]_i_ increases in HEK293 + GCaMP2 cells and astrocytes in the presence and absence of suramin (100 μM). The AUC of [Ca^2+^]_i_ increase was calculated during treatment with ATP for 1 min. n = 28–30 cells from three independent experiments; **p < 0.01 between control and the suramin-treated group at each ATP concentration, Bonferroni’s multiple comparison test after one-way ANOVA. (**e**) Representative maximum [Ca^2+^]_i_ projections of HEK293 + GCaMP2 for 2 min after mechanical stimulation with or without pharmacologic treatment. (**f**) Representative maximum [Ca^2+^]_i_ projection of an astrocyte (Fura2) and HEK293 + GCaMP2 (GCaMP2) during 2 min of treatment with 100 μM glutamate. (**g**) AUCs of [Ca^2+^]_i_ increases in HEK293 + GCaMP2 for 2 min after mechanical stimulation (CaW) or glutamate treatment with or without pharmacologic treatments. n = 28–30 cells from four independent experiments; **p < 0.01 versus CaW/(−), Bonferroni’s multiple comparison test after one-way ANOVA.

ATP release during the propagation of astrocyte calcium waves was investigated using an ATP sniffing cell, HEK293 cells expressing a fluorescent Ca^2+^ indicator protein, GCaMP2^25^. P_2Y1_ and P_2Y4_, but not P_2Y2_, P_2Y6_ or P_2X_ receptors are expressed in HEK293 cells and mediate ATP-induced [Ca^2+^]_i_ increases^26^. Meanwhile, cultured astrocytes express a variety of P_2X_ and P_2Y_ receptors, but their ATP-induced [Ca^2+^]_i_ increases are largely mediated by P_2Y_ receptors, except P_2×7_ receptor-mediated calcium influx in response to high concentrations of ATP^27^. Thus, it is assumed that HEK293 + GCaMP2 cells adjacent to astrocytes show [Ca^2+^]_i_ increases in response to extracellular ATP mediating astrocyte calcium waves. HEK293 + GCaMP2 cells were placed on top of Fura2-loaded astrocytes for simultaneous imaging of [Ca^2+^]_i_ in both cell types (Fig. 5a). HEK293 + GCaMP2 cells were seeded sparsely enough to avoid transmissions of [Ca^2+^]_i_ increases by their own gap junctions. Mechanical stimulation of astrocytes induced [Ca^2+^]_i_ increases in HEK293 + GCaMP2 cells, in areas equivalent to those of astrocyte calcium waves (Fig. 5b). To interpret these findings, the characteristics of [Ca^2+^]_i_ increases in HEK293 + GCaMP2 cells were analyzed. Transient [Ca^2+^]_i_ increases, which are characteristic of astrocyte [Ca^2+^]_i_ increases in the distal region, were induced in HEK293 + GCaMP2 cells by astrocyte calcium waves and ATP, as well as in astrocytes by ATP (Fig. 5c), indicating that the temporal dynamics of GCaMP2-expressing HEK293 cells is comparable to that of Fura2-loaded astrocytes. The EC_50_ values of HEK293 + GCaMP2 cells and astrocytes to ATP were 5.9 μM and 8.5 μM, respectively (Fig. 5d). Addition of 100 μM suramin significantly reduced the [Ca^2+^]_i_ increases in both cell types induced by ≤10 μM ATP. Thus, HEK293 + GCaMP2 cells and astrocytes respond to ATP in the same concentration range, and these responses are similarly sensitive to suramin, suggesting that HEK293 + GCaMP2 cells detect extracellular ATP with the same sensitivity as astrocytes.

Pharmacologic analysis of [Ca^2+^]_i_ increases by HEK293 + GCaMP2 cells during astrocyte calcium waves showed that these GCaMP2 responses were significantly reduced by suramin and apyrase, to 5% and 7%, respectively, confirming that they reflect astrocyte ATP releases (Fig. 5e and g). CBX, which abrogated calcium waves, reduced the GCaMP2 response to 7%, suggesting that interactions between astrocytes via gap junctions is essential for ATP release, and that extracellular ATP released during calcium waves is not likely due to mechanically-stimulated astrocytes alone. Ca^2+^-free medium, which abrogated the persistent [Ca^2+^]_i_ increases without affecting the peak and transient [Ca^2+^]_i_ increases, had no effect on GCaMP2 responses even in the proximal region, confirming that the propagation of the transient [Ca^2+^]_i_ increases was mediated by extracellular ATP and suggesting that the persistent [Ca^2+^]_i_ increases in the proximal region, which is larger than the transient [Ca^2+^]_i_ increases are not essential for ATP release. The finding, that ATP release is independent of [Ca^2+^]_i_ increase, was further strengthened by results showing that 100 μM glutamate induced robust [Ca^2+^]_i_ increases in astrocytes, but failed to induce GCaMP2 responses (Fig. 5f and g). These results indicate that [Ca^2+^]_i_ increases do not trigger astrocyte ATP release.

### Extracellular ATP diffusion during calcium waves

**Figure 6:**
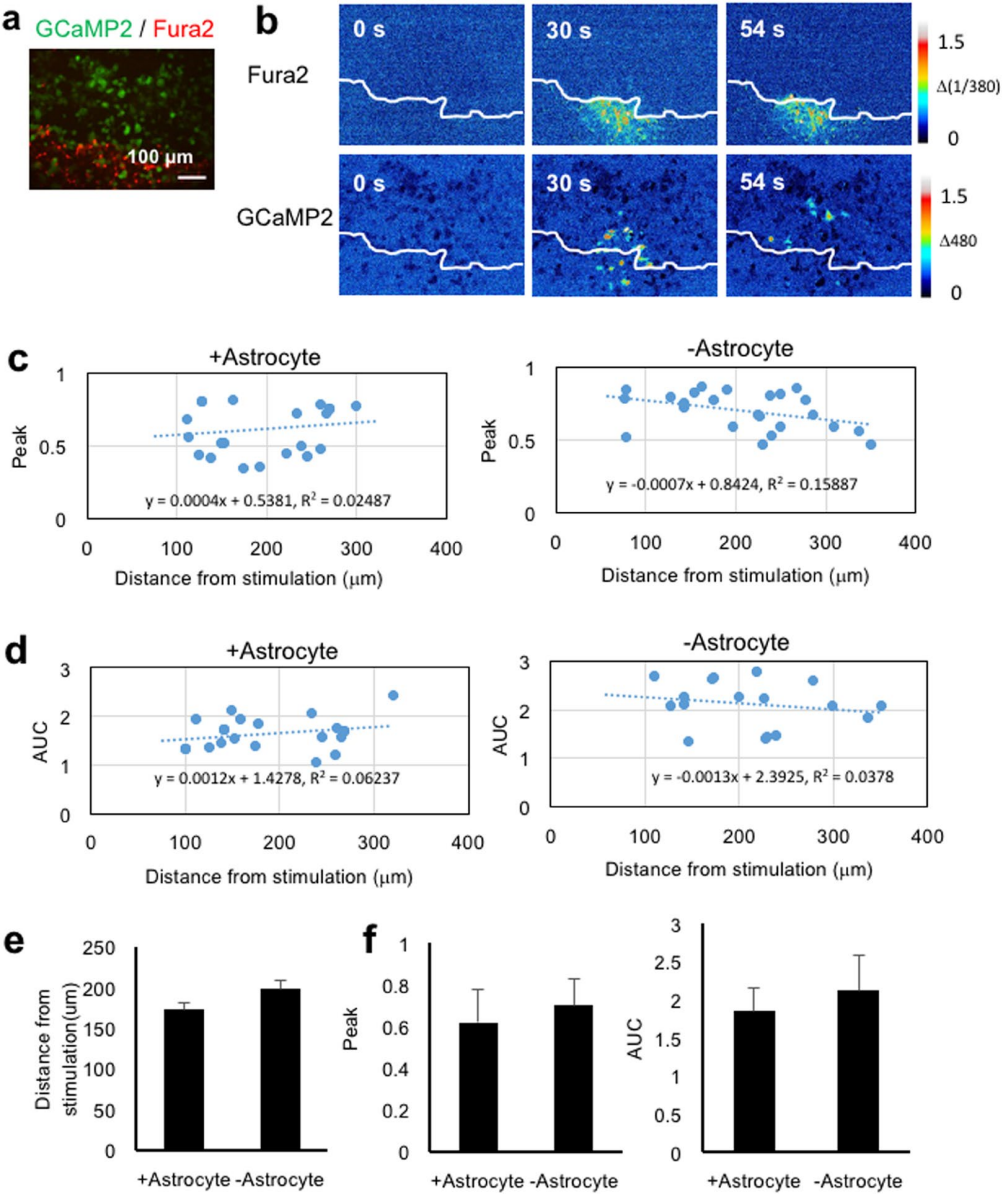
Distribution of extracellular ATP during calcium waves. Fura2-loaded astrocytes were scratched to create astrocyte-free areas, followed by seeding of HEK293 + GCaMP2 cells and mechanical stimulation of astrocytes close to the astrocyte-free area. (**a**) Representative fluorescence image of Fura2-loaded astrocytes (red) and HEK293 + GCaMP2 cells (green). (**b**) Representative images of [Ca^2+^]_i_ increases in astrocytes (Fura2) and HEK293 + GCaMP2 cells (GCaMP2) at 0, 30 and 54 sec after mechanical stimulation. The border of the astrocyte-free area in each panel is indicated by a white line. HEK293 + GCaMP2 cells showing 25–50% [Ca^2+^]_i_ increases were used in the following analyses: (**c**),(**d**) Distributions of peak amplitudes and AUCs of [Ca^2+^]_i_ increases in HEK293 + GCaMP2 cells on astrocytes (+Astrocyte) and on astrocyte-free areas (−Astrocyte), plotted against the distances between HEK293 + GCaMP2 cells and stimulated astrocytes. Regression lines are shown as dotted lines. The regression equation and correlation coefficient (R^2^) are included in each panel. Results are representative of four independent experiments. (**e**) Distances between HEK293 + GCaMP2 cells and stimulated astrocytes in the presence or absence of astrocytes. n = 32 cells from four independent experiments. (**f**) Peak amplitudes and AUCs of HEK293 + GCaMP2 cells in the presence and absence of astrocytes. n = 32 cells from four independent experiments. No significant difference was observed by Student’s t-test.

To monitor extracellular ATP diffusion during calcium waves, the GCaMP2 responses of astrocyte-free areas, generated by scratching astrocyte monolayers with a needle, were examined. Figure 6a is a representative fluorescence image of Fura2-loaded astrocytes after scratching and seeding HEK293 + GCaMP2 cells. Mechanical stimulation induced an astrocyte calcium wave and GCaMP2 responses in both astrocytes and astrocyte-free areas (Fig. 6b). HEK293 + GCaMP2 cells with [Ca^2+^]_i_ increases 25–50% of the highest [Ca^2+^]_i_ increase adjacent to stimulated astrocytes were subjected to further analysis for determining the influence of astrocytes on extracellular ATP diffusion. HEK293 + GCaMP2 cells with [Ca^2+^]_i_ increases >50% were excluded because these cells were mostly present in astrocyte-containing proximal regions. HEK293 + GCaMP2 cells with [Ca^2+^]_i_ increases <25% were also excluded, because of their sparse distribution. When peak amplitudes (Fig. 6C) and AUCs (Fig. 6d) of [Ca^2+^]_i_ increases in individual HEK293 + GCaMP2 cells showing 25–50% increases in [Ca^2+^]_i_ were plotted against the distance from the stimulated astrocyte, these HEK293 + GCaMP2 cells were found to be located approximately 100–300 μm from stimulated astrocytes, indicating that these cells were in the distal region. Their magnitude of [Ca^2+^]_i_ increase did not correlate with distance (R^2^ values were not significant) both in the presence and absence of astrocytes. The mean distances between stimulated astrocytes and HEK293 + GCaMP2 cells showing 25–50% increases in [Ca^2+^]_i_ and the amplitudes and AUC of their [Ca^2+^]_i_ increases were not affected by the presence of astrocytes (Fig. 6e and f). These results indicate that the GCaMP2 responses do not decline in the distal region, and are not affected by the presence of astrocytes. These findings therefore suggest that the transient [Ca^2+^]_i_ increases in the distal region is attributed to the diffusion of ATP released in the proximal region. Since HEK293 + GCaMP2 cell responses did not decline depending on the distance, the concentration of flowing extracellular ATP was likely constant, presumably due to continuous release, rather than diffusion of transiently-released ATP with dilution.

### ATP release Mechanisms in calcium waves

**Figure 7:**
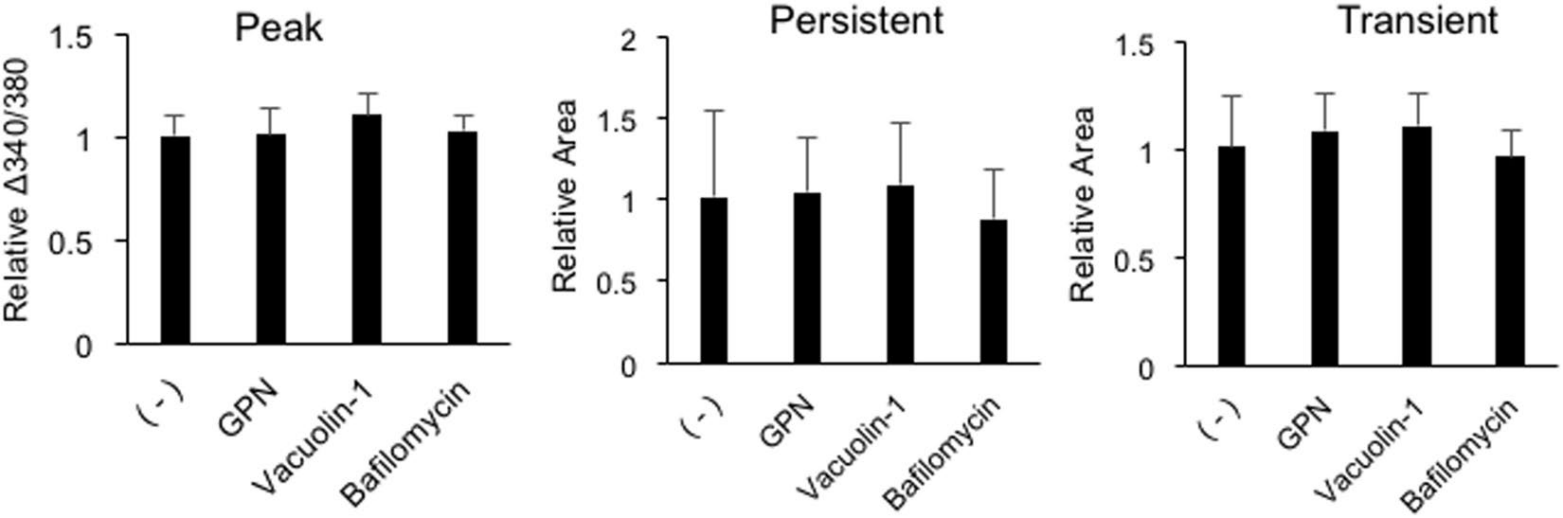
Effects of exocytosis inhibitors on calcium waves. Astrocytes were treated with 200 μM GPN or 2 μM bafilomycin for 1 h or with 10 μM vacuolin-1 for 10 min, followed by mechanical stimulation. Amplitudes of the peak [Ca^2+^]_i_ increases, and areas of the persistent and transient [Ca^2+^]_i_ increases are plotted normalized to control. n = 8 experiments. No significant difference was observed by one-way ANOVA.

**Figure 8:**
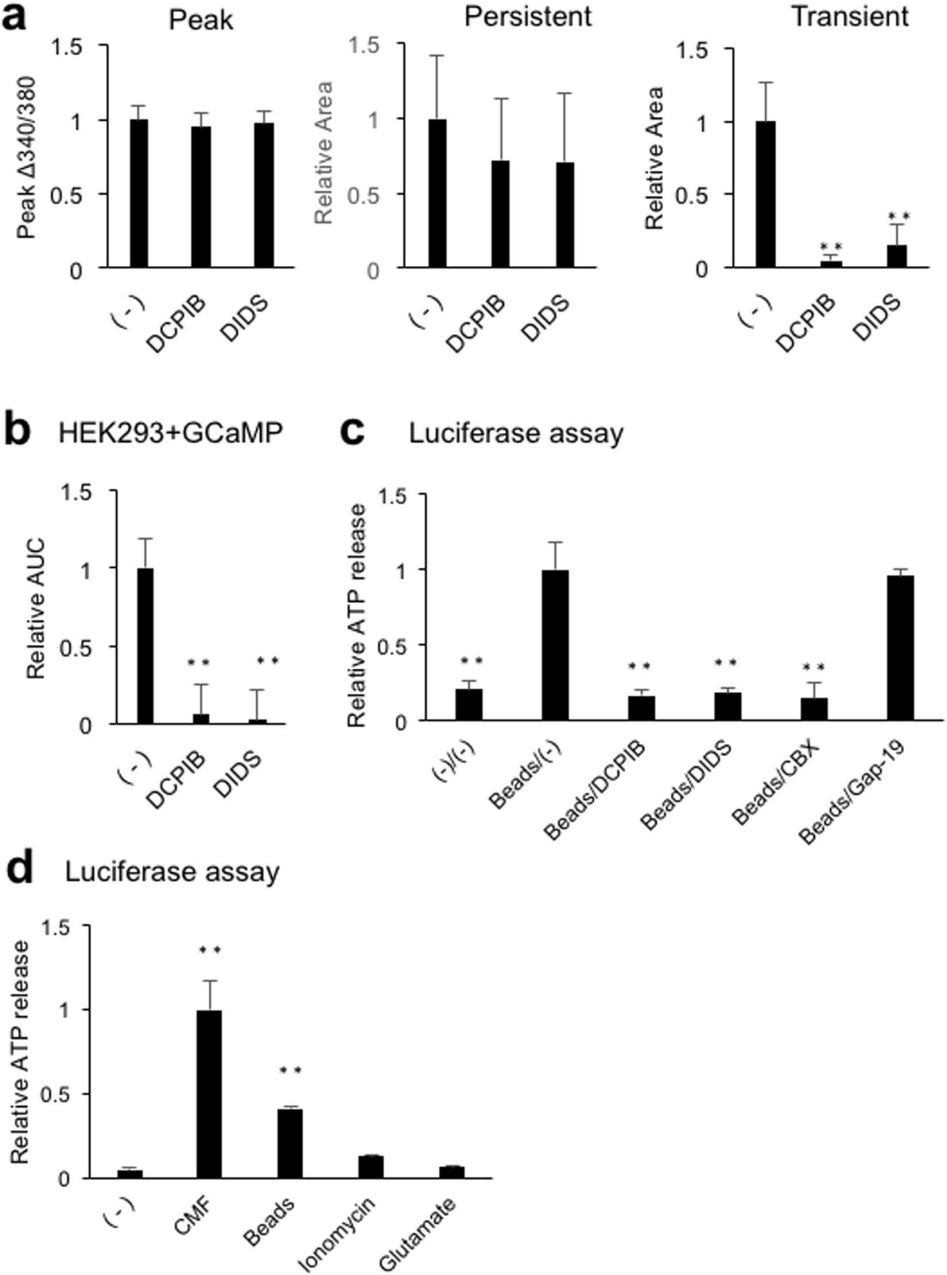
Effects of VRAC inhibitors on calcium waves and ATP release. Astrocytes were treated with 20 μM DCPIB or 200 μM DIDS for 10 min prior to mechanical stimulations. (**a**) Effects of VRAC inhibitors on amplitudes of the peak [Ca^2+^]_i_ increases and areas of the persistent and transient [Ca^2+^]_i_ increases, normalized to control. n = 8 experiments; **p < 0.01 versus (−), Bonferroni’s multiple comparison test after one-way ANOVA. (**b**) Effects of VRAC inhibitors on ATP release measured by HEK293 + GCaMP2 cells. AUCs of [Ca^2+^]_i_ increases in HEK293 + GCaMP2 cells for 2 min after mechanical stimulation, normalized to control. n = 32 cells from four independent experiments; **p < 0.01 versus (−), Bonferroni’s multiple comparison test after one-way ANOVA. (**c**) Effects of VRAC inhibitors on ATP release measured by luciferase assay. Cells were mechanically stimulated by dropping 0.02 g glass beads (30–50 μm in diameter); extracellular solution was collected 10 min later and used for luciferase assays. n = 3 wells; **p < 0.01 versus Beads/(−), Bonferroni’s multiple comparison test after one-way ANOVA. (**d**) Comparison of ATP release pathways measured by luciferase assays. Astrocytes were stimulated by CMF, beads, 100 μM ionomycin, and 1 mM glutamate. n = 3 wells; **p < 0.01 versus (−), Bonferroni’s multiple comparison test after one-way ANOVA.

ATP release mechanisms underlying calcium waves were explored pharmacologically. To assess the involvement of lysosome exocytosis, which was suggested to be the ATP release mechanism during electrically-induced calcium waves in cultured astrocytes^28^, astrocytes were treated with a cathepsin C substrate, 200 μM glycyl-L-phenylalanine 2-naphthylamide (GPN), or a cell permeable triadin, 10 μM vacuolin-1, which reduce lysosomes. In addition, cells were treated with 2 μM bafilomycin, an inhibitor of vesicular ATPase that depletes vesicles for exocytosis. None of these inhibitors had any effect on the components of calcium waves, indicating that neither lysosome nor conventional exocytosis was involved in ATP release during calcium waves (Fig. 7). The involvement of volume regulated anion channels (VRACs) was also assessed by incubation with the VRAC blockers, 20 μM 4-[(2-Butyl-6,7-dichloro-2-cyclopentyl-2,3-dihydro-1-oxo-1H-inden-5-yl)oxy]butanoic acid (DCPIB) and 200 μM diisothiocyano-2,2′-stilbenedisulfonic acid (DIDS). Neither of these reagents affected the peak or persistent [Ca^2+^]_i_ increases, whereas both significantly reduced the transient [Ca^2+^]_i_ increases to 4% and 14%, respectively (Fig. 8a), suggesting that VRAC blockers eliminate ATP release. The effects of these VRAC blockers on astrocyte ATP releases were examined using HEK293 + GCaMP2 cells and luciferase assays. Both DCPIB and DIDS significantly reduced the GCaMP2 responses, to 6% and 2%, respectively (Fig. 8b). In the luciferase assays, astrocyte ATP release was induced mechanically by dropping glass beads, which was reported to induce astrocyte calcium waves similar to the method of induction by glass pipettes^19^. Because ATP releases during calcium waves measured by HEK293 + GCaMP2 assays were eliminated by CBX, the effects of gap junction, gap junction hemichannel, and VRAC blockers were tested. DCBPI, DIDS and CBX significantly reduced beads-induced ATP release to 16%, 18% and 15%, respectively, whereas gap-19 had no effect (Fig. 8c). DCPIB was reported to inhibit Cx43 hemichannel^29^, however the effects of DCPIB on calcium waves and beads-induced ATP release are not mediated by Cx43 hemichannel, because both were not affected by the Cx43 hemichannel selective inhibitor, gap-19. These results indicate that VRAC blockers eliminate propagations of the transient [Ca^2+^]_i_ increases by blocking mechanically-induced ATP release and that gap junctions are involved in mechanically-induced ATP release.

Finally, ATP release by CMF, beads, ionomycin and glutamate were compared quantitatively. The amount of ATP released by beads was 43% that by CMF, whereas neither 100 μM ionomycin nor 1 mM glutamate induced detectable ATP release (Fig. 8d). These results indicate that mechanically-induced ATP release via VRAC is comparable to the ATP released by gap junction hemichannels, whereas [Ca^2+^]_i_ does not induce release of ATP.

## Discussion

The present study demonstrates that mechanically-induced astrocyte calcium waves consist of three components; (i) [Ca^2+^]_i_ increases in mechanically stimulated cells, which are independent of Ca^2+^ influx and IICR, (ii) fast propagation of persistent [Ca^2+^]_i_ increases in the proximal region, which are mediated by gap junctions and (iii) slow propagation of transient [Ca^2+^]_i_ increases in the distal region, which are mediated by extracellular ATP. These calcium waves also involve ATP release via VRAC activated by non-Ca^2+^ signal propagation via gap junctions in the proximal region.

The findings in the present study are in good agreement with those of previous studies. For example, the velocities of the calcium waves (22.6 ± 2.9 μm/sec in the proximal region and 9.3 ± 1.6 μm/sec in the distal region) were equivalent to those previously reported, 20 μm/sec.^1^ and 13.9 μm/sec.^30^; and the radii of the distal region (201 μm) were similar to those of previous reports, 204 μm^30^ and 180 μm^2^. Furthermore, the decline in calcium wave amplitudes and velocities during propagation^2^ and the discontinuous distribution of [Ca^2+^]_i_^16^ were shown in previous studies on astrocyte calcium waves. However, to our knowledge, this is the first study demonstrating distinct components of astrocyte calcium waves, and showing different contributions of gap junction and extracellular ATP to calcium wave propagation by characterizing these components.

We also found that the peak [Ca^2+^]_i_ increases were not affected by any pharmacologic inhibitors of [Ca^2+^]_i_ increase or propagation. The distinction we observed between the pharmacologic characteristics of peak [Ca^2+^]_i_ and adjacent propagating [Ca^2+^]_i_ increases were consistent with those of previous studies, which showed that a PLC and a SERCA inhibitor eliminated propagating [Ca^2+^]_i_ increases in the calcium waves of airway epithelial cells^31^ and smooth muscle cells^32^, respectively, without affecting the [Ca^2+^]_i_ increases in mechanically-stimulated cells. The lack of effect of the absence of extracellular Ca^2+^ on peak [Ca^2+^]_i_ increases excludes the possibility that touching with glass pipette tips induced the initial [Ca^2+^]_i_ increases by Ca^2+^ influx via mechanosensitive channels expressed in astrocytes^33^, or via physically-damaged plasma membranes. Since both omitting extracellular Ca^2+^ and inhibiting IICR did not affect increases in peak [Ca^2+^]_i_, mechanical stimulation may induce Ca^2+^ release from thapsigargin-insensitive calcium stores, which are not involved in IICR^34,35^.

The persistent [Ca^2+^]_i_ increases, which were sustained in the proximal region for several minutes, were larger and propagated faster than the transient [Ca^2+^]_i_ increases. Since these [Ca^2+^]_i_ increases were eliminated by IICR inhibitors and by omitting extracellular Ca^2+^, they may be shaped synergistically by IICR and Ca^2+^ influx. The induction of IP_3_ by mechanical stimulation has been demonstrated pharmacologically in many cell types including astrocytes^31,36^. Theoretical studies have suggested that IP_3_ mobilization through gap junctions is the main pathway of astrocyte calcium waves, and that the regenerative production of IP_3_ by Ca^2+^ dependent PLC subtypes drives the propagation^17,37^. Regenerative IP_3_ production likely underlies the persistent [Ca^2+^]_i_ increases and the gap junction-mediated propagation of calcium waves. It may also be essential for ATP release in the proximal region, whereas the IP_3_ production for the transient [Ca^2+^]_i_ increases, which was mediated by P_2Y_ receptors, was not amplified by Ca^2+^-dependent PLC or capable of inducing ATP release.

Omitting extracellular Ca^2+^ eliminated the persistent [Ca^2+^]_i_ increases, indicating that the difference between the persistent and transient [Ca^2+^]_i_ increases was due to Ca^2+^ influx. Our attempts to pharmacologically identify calcium channels responsible for Ca^2+^ influx have been unsuccessful. Purinergic inhibitors did not influence persistent [Ca^2+^]_i_ increases, whereas HEK293 + GCaMP2 cells detected extracellular ATP in the proximal region. These findings suggested that ATP occupies ATP receptors, but that ATP-induced IICR is occluded by the large persistent [Ca^2+^]_i_ increases in the proximal region. Omitting extracellular Ca^2+^ likely eliminated these large persistent [Ca^2+^]_i_ increases, allowing detection of ATP-induced IICR in the proximal region.

Because the transient [Ca^2+^]_i_ increases are abrogated by purinergic inhibitors and are insensitive to extracellular Ca^2+^, these increases have been attributed to ATP-induced IICR. The finding, that extracellular ATP in the distal region, as measured by HEK293 + GCaMP2 during calcium waves, was preserved even when astrocytes were removed by scratching, suggested that ATP is released within the proximal region and passively diffuses into the distal region as a continuous flow. These findings contradict a previous hypothesis, that ATP is released by a Ca^2+^-dependent regenerative process during propagation^14^. The lack of a regenerative process likely accounts for the lower velocity in the distal region. That is, [Ca^2+^]_i_ increases in the proximal region propagate rapidly and effectively by direct intracellular communications via gap junctions and regenerative IP_3_ production. In contrast, [Ca^2+^]_i_ increases in the distal region propagate slowly and passively by diffusion of ATP.

HEK293 + GCaMP2 cells, which are equivalent in sensitivity and pharmacologic properties to astrocytes in detecting extracellular ATP, were used in the present study to measure ATP release during calcium waves. Our findings, that purinergic inhibitors abolished GCaMP2 responses without affecting astrocyte [Ca^2+^]_i_ increases in the proximal region, suggested that extracellular ATP transmits [Ca^2+^]_i_ increases between astrocytes and HEK293 + GCaMP2. Moreover, the ability of CBX to abolish GCaMP2 responses suggests the involvement of gap junctions in ATP release. These findings, together with results showing that exogenous expression of connexin potentiated ATP release via anion channels^38^, indicate that VRAC is activated by a signal mediated by gap junctions. This signal is not likely to be [Ca^2+^]_i_, because (i) Ca^2+^-free medium reduced proximal [Ca^2+^]_i_ increases without affecting ATP release measured by GCaMP2 responses; (ii) glutamate induced robust [Ca^2+^]_i_ increases in astrocytes but not in adjacent HEK293 + GCaMP2 cells, and (iii) glutamate and ionomycin did not induce ATP releases detectable by luciferase assays. The Ca^2+^ independent ATP release during astrocyte calcium waves is consistent with previous imaging of extracellular ATP by luciferase^36^. In addition, Bergman glia were shown to release glutamate via DIDS-sensitive anion channels following optogenetic stimulation^39^, suggesting the possibility that depolarization and/or associated movement of ions trigger DIDS-sensitive release of anionic molecules.

Because ATP in astrocytes is involved in various intercellular communications, these cells are equipped with multiple ATP release mechanisms, including exocytosis, gap junction hemichannels, P_2 × 7_ receptor and VRAC^40^. In the present study, luciferase assays showed substantial ATP release via gap junction hemichannels and VRAC, but not via exocytosis. GPN inhibition of lysosome exocytosis was reported to suppress ATP release during electrically-induced astrocyte calcium waves, which were potentiated by metabolic inhibition^41^. The lack of exocytosis in the present study may reflect differences in stimulation methods or culture conditions that affect metabolism, or the slow and ineffective progression of astrocyte exocytosis, as shown by total internal reflection fluorescence imaging^42^.

The protein, leucine-rich repeat containing 8 family member A (LRRC8A) was identified as a DCPIB-sensitive VRAC^43,44^. The involvement of LRRC8A in ATP release during astrocyte calcium wave propagation was examined using siRNA, however no significant influence of knocking-down LRRC8A was found (data not shown), suggesting the involvement of other unidentified VRAC genes. LRRC8A was found to be essential for glutamate release by astrocytes in response to ATP treatment or hypo-osmotic conditions^45^. However, most DCPIB-sensitive anion conduction in astrocytes has been associated with the release of glutamate, not ATP, although some glutamate release is triggered by ATP^45,46^. Mechanically-induced ATP release is thought to be due to other VRAC subtypes designated as maxi-anion channels^47^, which are also activated by mechanical stimulation including swelling by hypo-osmotic conditions. The gene encoding maxi-anion channels has not yet been identified. These channels are thought to possess a large pore, suitable for the permeation of ATP, which has a larger effective radius than glutamate^48^. Thus, the VRAC genes involved in astrocyte calcium wave are yet to be determined.

The present study found that a single mechanical stimulation of an individual astrocyte induced ATP release via VRAC from adjacent astrocyte networks connected by gap junctions. This process may represent the initiation and/or propagation of pathological processes following brain injuries. Microglia co-cultured with astrocytes respond to adjacent astrocyte calcium waves by increasing [Ca^2+^]_i_^49^ or releasing IL1β^50^. *In vivo* microglia adjacent to focal insults to brain parenchyma undergo structural changes in response to ATP released by astrocytes via a mechanism involving gap junctions^51^. This pathological activation of microglia is likely due to astrocyte release of ATP during calcium waves. Furthermore, blockers of P_2Y_ receptors, including suramin^52,53^, gap junctions^54^ and VRAC^55^ are neuroprotective after brain injury, strengthening the pathological implications of astrocyte ATP release. DCPIB has been reported to attenuate inflammatory responses after brain injury^56^. Further investigations based on the results of this study may identify the mechanically-induced signaling molecules, which propagate via gap junctions, and subsequent cascades for activating VRAC after mechanical stimulation, providing new insights into mechanisms of brain injury and possibly identifying therapeutic targets.

This detailed analysis of a classical model has revealed novel aspects of astrocyte physiology, especially the different contributions of two major pathways of calcium waves, gap junctions and extracellular ATP. The physiological and pathological implications of [Ca^2+^]_i_ dynamics and purinergic signaling of astrocytes remain incompletely understood. Addressing the mechanisms of action and functions of astrocyte [Ca^2+^]_i_, including calcium waves, calcium oscillation, and spontaneous [Ca^2+^]_i_ increases using cell culture models, remains important in basic and clinical research.

## Methods

### Cell culture

All animal experiments were approved by the Institutional Animal Care and Use Committee of Kobe University (Permission number: 25–20) and performed according to the Kobe University Animal Experimentation Regulations. Rats of either sex aged 0–2 postnatal days were sacrificed, and their cerebral cortices were removed and chopped into small pieces in Hank’s Balanced Salt Solution (Thermo Fisher Scientific, Franklin, MA). Cell preparations were incubated with 0.25% trypsin (Worthington Biochem, Freehold, NJ), with occasional agitation, at 37 °C for 20 min. DNase (Sigma-Aldrich, St. Louis, MO) was added to a final concentration of 0.1% and the cell preparations were incubated at room temperature for 5 min. The samples were transferred to Dulbecco’s Modified Eagle’s Medium containing 10% fetal bovine serum (FBS, Nichirei Biosciences, Tokyo, Japan) and antibiotics (50 μg/ml penicillin and 50 μg/ml streptomycin) and dispersed by pipetting. The resulting cells were seeded at 2.5 × 10^4^ cells/cm^2^ in 75 cm^2^ flasks and cultured in the presence of 5% CO_2_ at 37 °C for 1–2 weeks. The mixed brain cell cultures were shaken to remove less-adherent cells and treated with trypsin. The resulting astrocyte preparations were seeded at 2.5 × 10^4^ cells/cm^2^ on 12 mm coverslips, which were incubated overnight with 1 μg/ml poly-D-lysine (Sigma) for imaging analysis; or in wells of 96 well plates for luciferase assays. Astrocytes were subjected to experiments 1–3 weeks after seeding.

HEK293 cells (ATCC, Manassas, VA) were maintained in DMEM/F12 containing 10% FBS and antibiotics, and subcultured by treating with 10 mM EDTA/PBS. Cells were transfected with pN1-G-CaMP2 (RIKEN, Saitama, Japan) using Geneporter (Genlantis, San Diego, CA) and screened with 2 mg/ml G418 (Roche, Indianapolis, IN) and by fluorescence imaging to obtain stable transfectants expressing GCaMP2.

### Calcium imaging

Astrocytes were washed three times with Basal Salt Saline (BSS; 129 mM NaCl, 4 mM KCl, 1 mM MgCl_2_, 2 mM CaCl_2_, 10 mM D-glucose, 10 mM Hepes pH 7.4) and incubated with 7.5 μM Fura2-AM (Dojin, Kumamoto Japan) at 30 °C for one hour. After three additional washes with BSS, the cells were maintained in BSS containing 100 μM sulfinpyrazone (Sigma), which inhibits Fura2 sequestration and secretion^57^, at room temperature until imaging experiments. HEK293 + GCaMP2 cell suspensions were added to Fura2-loaded astrocytes in BSS, and cocultured for more than two hours at room temperature. Fluorescence images were obtained using an inverted microscope, IX70 (Olympus, Tokyo, Japan) equipped with a filter exchanger (MAC5000; Ludl Electronic Products, Hawthorne, NY) and a cooled-CCD camera, Orca-R2 (Hamamatsu Photonics, Hamamatsu, Japan). The objective lens was UApo/340 20×/0.70w (Olympus). A 0.5x camera adaptor U-TV0.5XC (Olympus) was inserted before the camera. For Fura2-based calcium imaging, a set of F340 (Ex/DM/Em = 340/500/535) and F380 (Ex/DM/Em = 380/500/535) images were obtained every three seconds. For simultaneous calcium imaging of Fura2-loaded astrocytes and HEK293 + GCaMP2 cells, a set of F380 and F480 (Ex/DM/Em = 480/500/535) images were obtained every three seconds. Astrocytes were mechanically stimulated by touching them with 1–10 MΩ tips of a glass pipette (G150F-3, Warner Instruments, Hamden, CT), fabricated using P-87 (Sutter Instrument, Novato, CA). To maintain a constant stimulation force, contact by the tip was measured by input impedance using an amplifier (CEZ-2400; Nihon Kohden, Hyodo, Japan), and its approach was stopped immediately upon any increase in input impedance. The tip remained in contact during calcium wave propagation, because the waves were unaffected regardless of tip withdrawal.

### Data analysis

Shading of fluorescence images was corrected by dividing the value of each pixel by the corresponding value in a control image. For Fura2-based calcium imaging, the Δ340/380 ration was calculated as $\frac{{\rm{F}}340-{\rm{BG}}1}{{\rm{BG}}340-{\rm{BG}}1}/\frac{{\rm{F}}380-{\rm{BG}}2}{{\rm{BG}}380-{\rm{BG}}2}$, where BG1 and BG2 were the fluorescence images obtained in the absence of excitation using filter sets for F340 and F380, respectively, and BG340 and BG380 (control image) were the F340 and F380 images of 10 μM Fura2 potassium salt solution, respectively. For simultaneous imaging of GCaMP2 and Fura2, ${\rm{F}}^{\prime} 480=\frac{{\rm{F}}480-{\rm{BG}}}{{\rm{BG}}480-{\rm{BG}}}$, where BG was the fluorescence image obtained in the absence of excitation using a filter set for F480, BG480 was the F480 image of 10 μM fluorescein, and ${\rm{F}}^{\prime} 380\,=\,\frac{{\rm{F}}380-{\rm{BG}}}{{\rm{BG}}380}$ was used to calculate $\Delta 480=\frac{F\text{'}480}{{\rm{Fo}}480}$ where Fo480 was the average of five F′480 images before stimulation and $\Delta (1/380)=1/\frac{F\text{'}380}{{\rm{Fo}}380}$, where Fo380 was the average of five F′380 images before stimulation

### Luciferase assay

Astrocytes were equilibrated with BSS at 30 °C for longer than 15 min, pretreated with inhibitors for 10 min, and stimulated by incubation with 100 μl BSS containing inhibitor and stimulus for 10 min. A 50 μl aliquot of the extracellular solution used for stimulation was mixed with 50 μl assay reagent (ATP Bioluminocount Assay Kit CLS II, Sigma-Aldrich) and luciferase activity was measured using a luminometer (GLOMAX 20/20; Promega, Fitchburg, WI).

### Materials

18β-Glycyrrhetinic acid, apyrase, ATP, thapsigargin, and U73122 were obtained from Sigma-Aldrich. DCPIB was obtained from Tocris (Bristol, UK); DIDS was from Tokyo Chemical Industry Co. Ltd (Tokyo, Japan); and GPN, vacuolin-1 and Xestospongin C were from Santa Cruz Biotechnology (Dallas, TX). Gap-19 was synthesized (HPLC grade) by MBL (Kyoto, Japan). All other chemicals were from Nacalai (Kyoto, Japan).

### Statistical analysis

The values are expressed as mean values ± SD. Differences among the values were analyzed using statistical methods mentioned in Figure Legends and differences with probability (p) values < 0.05 were considered significant.
